# Multiple sclerosis-disease modifying therapies affect humoral and T-cell response to mRNA COVID-19 vaccine

**DOI:** 10.3389/fimmu.2022.1050183

**Published:** 2022-12-01

**Authors:** Federica Dominelli, Maria Antonella Zingaropoli, Matteo Tartaglia, Eeva Tortellini, Mariasilvia Guardiani, Valentina Perri, Patrizia Pasculli, Federica Ciccone, Leonardo Malimpensa, Viola Baione, Anna Napoli, Aurelia Gaeta, Miriam Lichtner, Antonella Conte, Claudio Maria Mastroianni, Maria Rosa Ciardi

**Affiliations:** ^1^Department of Public Health and Infectious diseases, Sapienza, University of Rome, Rome, Italy; ^2^Department of Human Neurosciences, Multiple Sclerosis Centre, Sapienza, University of Rome, Rome, Italy; ^3^Department of Molecular medicine, Sapienza, University of Rome, Rome, Italy; ^4^Infectious Diseases Unit, Santa Maria Goretti Hospital, Sapienza, University of Rome, Latina, Italy; ^5^Department of Neurosciences Mental Health and Sensory Organs, Sapienza University of Rome, Rome, Italy; ^6^Scientific Hospitalization and Treatment Institute, Neuromed Mediterranean Neurological Institute, Pozzilli, Italy

**Keywords:** SARS-CoV-2 mRNA vaccine, T-cell, MS, DMTs, flow-cytometry, BAFF, April, CD40L

## Abstract

**Background:**

The mRNA vaccines help protect from COVID-19 severity, however multiple sclerosis (MS) disease modifying therapies (DMTs) might affect the development of humoral and T-cell specific response to vaccination.

**Methods:**

The aim of the study was to evaluate humoral and specific T-cell response, as well as B-cell activation and survival factors, in people with MS (pwMS) under DMTs before (T0) and after two months (T1) from the third dose of vaccine, comparing the obtained findings to healthy donors (HD). All possible combinations of intracellular IFNγ, IL2 and TNFα T-cell production were evaluated, and T-cells were labelled “responding T-cells”, those cells that produced at least one of the three cytokines of interest, and “triple positive T-cells”, those cells that produced simultaneously all the three cytokines.

**Results:**

The cross-sectional evaluation showed no significant differences in anti-S antibody titers between pwMS and HD at both time-points. In pwMS, lower percentages of responding T-cells at T0 (CD4: p=0.0165; CD8: p=0.0022) and triple positive T-cells at both time-points compared to HD were observed (at T0, CD4: p=0.0007 and CD8: p=0.0703; at T1, CD4: p=0.0422 and CD8: p=0.0535). At T0, pwMS showed higher plasma levels of APRIL, BAFF and CD40L compared to HD (p<0.0001, p<0.0001 and p<0.0001, respectively) and at T1, plasma levels of BAFF were still higher in pwMS compared to HD (p=0.0022).

According to DMTs, at both T0 and T1, lower anti-S antibody titers in the depleting/sequestering-out compared to the enriching-in pwMS subgroup were found (p=0.0410 and p=0.0047, respectively) as well as lower percentages of responding CD4+ T-cells (CD4: p=0.0394 and p=0.0004, respectively). Moreover, the depleting/sequestering-out subgroup showed higher percentages of IFNγ-IL2-TNFα+ T-cells at both time-points, compared to the enriching-in subgroup in which a more heterogeneous cytokine profile was observed (at T0 CD4: p=0.0187; at T0 and T1 CD8: p =0.0007 and p =0.0077, respectively).

**Conclusion:**

In pwMS, humoral and T-cell response to vaccination seems to be influenced by the different DMTs. pwMS under depleting/sequestering-out treatment can mount cellular responses even in the presence of a low positive humoral response, although the cellular response seems qualitatively inferior compared to HD. An understanding of T-cell quality dynamic is needed to determine the best vaccination strategy and in general the capability of immune response in pwMS under different DMT.

## Introduction

In the last two years, the coronavirus disease 2019 (COVID-19), caused by Severe Acute Respiratory Syndrome CoronaVirus-2 (SARS-CoV-2), has emerged with a severe global health impact and difficult clinical management (1, 2). SARS-CoV-2 vaccines with different designs have been approved and authorized in many countries, Italy included, and vaccine campaigns have been launched (3). Among them, the mRNA vaccine mRNABNT162b2 (Comirnaty^®^) has been widely employed in the Italian population (4). Several studies showed that mRNA vaccines help protect from severe COVID-19 disease, hospitalization and death in immunocompetent individuals and in frail populations (5, 6). However, multiple sclerosis (MS) disease modifying therapies (DMTs) might affect COVID-19 disease severity as well as the development of humoral and cellular immunity after SARS-CoV-2 exposure or vaccination (7, 8). Indeed, Sormani et al. (9) showed a propensity toward a more severe COVID-19 disease in people with MS (pwMS) under certain DMTs, such as anti-CD20 treatments.

MS is an inflammatory demyelinating disease affecting the central nervous system (CNS), thought to result from the interaction of genetic and environmental factors that remain only partially understood (10). Several DMTs have been developed and are now currently available (11). These drugs act at different levels on the immune system causing (I) depletion and/or cytolysis of immune cells, such as anti-CD20 humanized monoclonal antibody (ocrelizumab), anti-CD52 monoclonal antibody that depletes CD52+ T- and B-cells (alemtuzumab) and purine analogue that interferes with DNA synthesis inducing prolonged lymphocyte depletion (cladribine), and (II) an impairment of immune cell migration, such as α4-integrin antibody that prevents lymphocytes blood–brain barrier (BBB) crossing (natalizumab) and a non-selective sphingosine 1 phosphate (S1P) receptor modulator that prevents lymphocyte egress from lymph nodes (fingolimod) (2, 11). Despite the remarkable effectiveness, DMTs are usually associated to an increased risk of infections, such as tuberculosis, hepatitis B, John Cunningham (JC) virus, herpes viruses reactivation (12–20) and an attenuation of responses to vaccination, that seems to be related to the drug’s mode of action (21–24).

B-cell activating and survival factors, like B-cell activating factor (BAFF), A-proliferation inducing ligand (APRIL) and CD40L ligand (CD40L), are mainly implicated in B-cell survival, proliferation and antibody production and T-cell dependent and independent antibody class switching (25–27). After vaccination their concentrations increases enhancing B-cell activation (28, 29), and their expression is a prerequisite for activation of adaptive immune response to vaccination, while their absence may result in a reduced magnitude of response (27). Being involved in B-cell differentiation and survival, the three cytokines are target for immune modulation in the context of vaccine design and have been recently studied as molecular adjuvants to improve vaccine outcome (30).

The aim of the study was to evaluate humoral and specific T-cell response, as well as B-cell activating and survival factors in pwMS under different DMTs.

## Materials and methods

### Ethics statement

The study was approved by Ethics Committee of Policlinico Umberto I, Sapienza University of Rome (protocol numbers 0062/2022). All patients gave written consent for participation in the study.

### Study design and participants

To evaluate humoral and specific T-cell response to mRNABNT162b2 (Comirnaty^®^) vaccine, pwMS under different DMTs and age- and sex-matched healthy donors (HD) were enrolled. Prior history of symptomatic SARS-CoV-2 infection was considered as exclusion criterion. Both pwMS and HD received two dose of mRNABNT162b2 (Comirnaty^®^) vaccine according to schedule proposed by the current Italian national vaccination program (4). For both groups, two time-points were considered: before (T0) and after two months from the third dose of mRNABNT162b2 vaccine (T1).

All enrolled pwMS were stratified according to the drug’s mechanism of action on peripheral blood cells into two subgroups: depleting/sequestering-out, including those patients treated with alemtuzumab, cladribine, fingolimod and ocrelizumab, and enriching-in, including those patients treated with natalizumab. The blood samples from pwMS treated with cladribine, ocrelizumab or alemtuzumab were taken at least 3 months after last drug administration. The differences in humoral and specific T-cell response as well as in B-cell activating and survivor factors, among the two subgroups were evaluated.

### SARS-CoV-2 anti-N and anti-S antibodies

To exclude possible pre-exposure to asymptomatic natural SARS-CoV-2 infection, specific SARS-CoV-2 anti-Nucleocapsid (N) antibodies were measured on serum using the KT-1032 EDI TM Novel Coronavirus COVID-19 IgG Enzyme Linked Immunosorbent Assay (ELISA) Kit (Epitope Diagnostics, Inc. 7110 Carroll Rd, San Diego, CA 92121, USA) and performed according to the manufacturer’s instructions. The average value of the absorbance of the negative control is less than 0.25 optical density (OD), and the absorbance of the positive control is not less than 0.30 OD.

Specific SARS-CoV-2 total anti-Spike antibodies were evaluated in serum, for all time-points, using a commercial chemiluminescence immunoassay (CLIA) (The DiaSorin Liaison SARS-CoV-2 TrimericS IgG; DiaSorin S.p.A) according to manufacturer’s instructions. The test detects SARS-CoV-2 Spike S1/S2 protein specific IgG antibody levels, expressed in binding antibody unit (BAU/ml) according to World Health Organization international Reference Standard (NIBSC code. 20/268). A positive serologic response was defined as having detectable IgG antibodies against SARS-CoV-2 over the cut-off value of 33.8 BAU/ml.

### T-cell stimulation with SARS-CoV-2 specific peptide libraries

T-cell specific response was assessed using a multiparametric flow cytometry after overnight stimulation with SARS-CoV-2 peptide libraries on isolated peripheral blood mononuclear cells (PBMCs), as previously described (12, 21, 31). Pools of lyophilized peptides, consisting mainly of 15-mer sequences with 11 amino acids overlap, covering the immunodominant sequence domains of the Spike glycoprotein (S) (GenBank MN908947.3, Protein QHD43416.1) and the Nucleocapsid phosphoprotein (N) (GenBank MN908947.3, Protein QHD43423.2) of SARS-CoV- 2 were purchased from Miltenyi Biotec. Specifically, PepTivator SARS-CoV-2 Prot_S1 covered the N-terminal S1 domain of the spike protein (amino acids [aa] 1–692). PepTivator SARS-CoV-2 Prot_S covered selected immunodominant sequence domains of the spike protein (aa 304–338, 421–475, 492–519, 683–707, 741– 770, 785–802, and 885–1273). PepTivator SARS-CoV-2 Prot_N covered the complete sequence of the N phosphoprotein of SARS-CoV-2. For each patient, an unstimulated and a positive phytohemagglutinin (PHA) 5μg/ml control was also included. Brefeldin A at a final concentration of 5μg/ml was added in the culture after 1 hour of incubation.

PBMCs were stained with an appropriate combination of fluorochrome-conjugated antibodies (PacificBlue-conjugated anti-CD45, APC-Cy7-conjugated anti-CD4, APC-conjugated anti-CD8, BioLegend, San Diego). Fix/Perm solution (BioLegend, San Diego) was used prior intracellular staining (FITC-conjugated anti- IFNγ, PerCp-Cy 5.5-conjugated anti-TNFα and PE-Cy7-conjugated anti-IL2, BioLegend, San Diego), according to manufacturer’s instructions. Fixable viability kit (Zombie Aqua™ BioLegend, San Diego) was used to exclude dead cells. Samples were acquired using MACSQuant (Miltenyi Biotec, Germany) and analyzed using FlowJo™ v10.8.1 software. Specifically, cytokine background obtained from the unstimulated condition was subtracted to the stimulated ones. All possible combinations of intracellular expression of IFNγ, IL2 and TNFα in cytokine-producing T-cells were evaluated using the Boolean gate. “Responding T-cells” were defined as those cells that produce any of IFNγ, IL2 and TNFα, while “triple-positive T-cells” were defined as those simultaneously producing all three cytokines. Display and analysis of the different cytokine combinations was performed with SPICE v6.1.

### Measurement of BAFF, APRIL and CD40L

**Figure 1:**
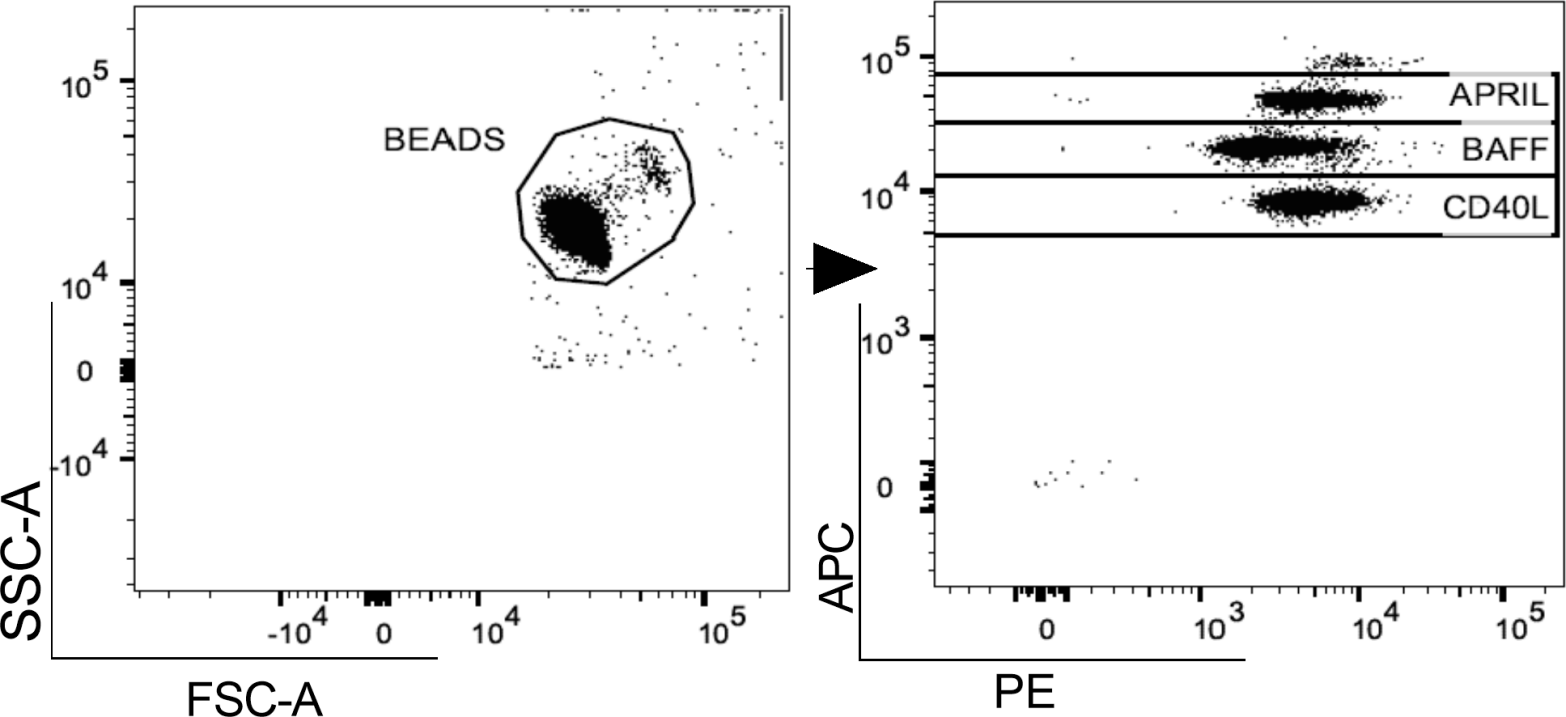
Gating strategy. A. Representative flow cytometry plot for the evaluation of plasma APRIL, BAFF and CD40L levels after bead-based multiplex assay panel. APRIL, A-proliferation inducing ligand; BAFF, B-cell activating factor; CD40L, CD40 ligand.

In both pwMS and HD, plasma levels of BAFF, APRIL and CD40L were measured using a commercial cytometric bead-based multiplex panel immunoassay (CBA) (BioLegend, San Diego), acquired using MACSQuant (Miltenyi Biotec, Germany) and analyzed using FlowJo™ v10.8.1 software (**Figure 1**). B-cell activating and survival factors were expressed as plasma concentration (pg/ml).

### Statistical analyses

All data are reported as median and interquartile range (IQR). Differences between pwMS and HD were assessed using a two-tailed Mann-Whitney test for quantitative variables. Differences among pwMS subgroups and HD were assessed using a non-parametric Kruskal-Wallis test with Dunn’s multiple comparison post-test for quantitative variables. Two-point longitudinal assessment was performed using a non-parametric Wilcoxon test. Results were considered statistically significant if the p value was <0.05. Statistical analyses were performed using GraphPad Prism 9. Finally, distributions of different cytokine combinations were performed by the nonparametric Wilcoxon rank test using SPICE, distributed by the National Institute of Allergy and Infectious Diseases, NIH.

## Results

### Study population

From October 2021 to June 2022, 18 pwMS (female/male: 12/6; 43 [35-56] years) and 18 HD (female/male: 13/5; 30 [30-53] years) were enrolled (**Table 1**). All pwMS were under DMTs and the median time (IQR) from starting the current treatment was of 3 [2-4] years. As reported in **Table 1**, among pwMS 5.5% were alemtuzumab-treated, 11.1% cladribine-treated, 11.1% fingolimod-treated, 33,3% natalizumab-treated and 38.9% ocrelizumab-treated. Given that, we stratified pwMS according to the drug’s mechanism of action on peripheral blood immune cells into two subgroups: depleting/sequestering-out (n=12; female/male: 7/5; 46 [35-57] years) and enriching-in (n=6; female/male: 5/1; 40 [34-44] years) (**Table 1**).

**Table 1:** Demographic and clinical features of study population.

	HD	pwMS	depleting/sequestering-out	enriching-in
Female/Male	13/5	12/6	7/5	5/1
Age, median (IQR)	30 (35–53)	43 (35–56)	46 (35–57)	40 (34–44)
Years of disease, median (IQR)	–	7 (3–14)	7 (5–15)	5 (1–9)
EDSS, median (IQR)	–	3 (1–4)	3 (1–6)	2 (1–3)
Previous MS treatment (yes/no)	–	5/13	3/9	2/6
Years of current treatment, median (IQR)	–	2 (2–4)	3 (2–4)	1 (1–4)
Current MS treatment	–			
alemtuzumab (n)		1		
cladribine (n)	–	2		
fingolimod (n)	–	2		
natalizumab (n)	–	6		
ocrelizumab (n)	–	7		

MS, multiple sclerosis; pwMS, people with multiple sclerosis; HD, healthy donors; n, number; IQR, interquartile range; EDSS, expanded disability status scale.

### The cross-sectional evaluation of humoral and specific T-cell response, and B-cell activating and survival factors

The cross-sectional evaluation of humoral and specific T-cell response, as well as B-cell activating and survival factors was performed at T0 comparing 18 pwMS (female/male: 12/6; 43 [35-56] years) and 12 HD (female/male: 8/4; 42 [33-53] years), and at T1 comparing 16 pwMS (female/male:11/5; 42 [34-49] years) and 15 HD (female/male: 12/3; 38 [30-52] years).

The evaluation of specific SARS-CoV-2 anti-N antibodies performed both T0 and T1 showed negative results for all enrolled pwMS and HD.

Overall, a positive serological response to vaccination was observed in 77.8% (14/18) and 88.0% (14/16) of enrolled pwMS, at T0 and T1, respectively. Conversely, a positive serological response at both time-points in 100% (12/12 and 15/15, respectively) of enrolled HD was found.

**Figure 2:**
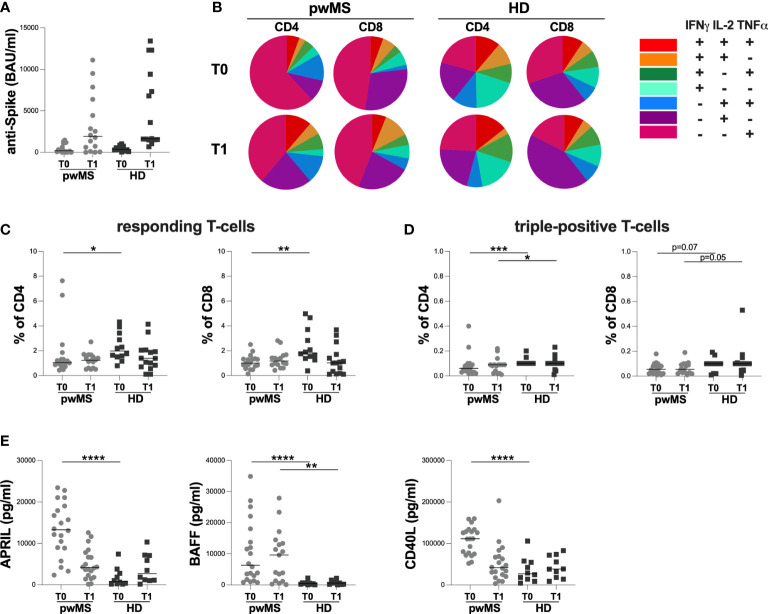
Cross-sectional evaluation of humoral and specific T-cell response in pwMS and HD, and overview of cytokine-producing T-cells. **(A)** The evaluation of anti-S antibody titers in pwMS and HD. **(B)** Overview of intracellular IFNγ,IL2 and TNFα production by CD4+ and CD8+ T-cells at T0 and at T1 in pwMS and HD. **(C)** Evaluation of percentage in responding CD4+ and CD8+ T-cells in pwMS and HD. **(D)** Evaluation of percentage in triple-positive CD4+ and CD8+ T-cells in pwMS and HD. **(E)** Evaluation of plasma levels of APRIL, BAFF and CD40L in pwMS and HD. APRIL, A-proliferation inducing ligand; BAFF, B-cell activating factor; CD40L, CD40 ligand; pwMS, people with multiple sclerosis; HD, healthy donors; T0, before third dose of vaccine; T1, after two months form third dose of vaccine. *p < 0.05; **p < 0.01; ***p < 0.001; ****p < 0.0001.

The cross-sectional evaluation of anti-S antibody titers showed no statistically significant differences between pwMS and HD at both time-points (T0: 199 [60-1120] and 369 [189-700.50] BAU/ml, respectively; T1: 1930 [225-5895] and 1660 [1520-9400] BAU/ml, respectively) (**Figure 2A**).

As reported in **Figure 2B**, at both time-points, we observed a different T-cell subset distribution in pwMS and HD, with a more heterogeneous production of the three cytokines in the latter (**Figure 2B**).

At T0, a lower percentage of responding CD4+ and CD8+ T-cells in pwMS compared to HD was observed (CD4: 1.04 [0.85-1.44] and 1.98 [1.52-3.29], respectively, p=0.0165; CD8: 1.00 [0.71-1.34] and 1.82 [1.42-3.48], respectively, p=0.0022) (**Figure 2C**). Otherwise, at T1, not statistically differences in the percentage of responding T-cells were found (CD4:1.23 [0.60-1.63] and 1.39 [0.70-2.05], respectively; CD8: 1.17 [0.86-1.56] and 1.02 [0.17-1.70], respectively) (**Figure 2C**).

At both T0 and T1, lower percentages of triple-positive T-cells were seen, although only a trend for CD8+ T-cells was observed (CD4: 0.06 [0.03-0.09] and 0.10 [0.10-0.10], respectively, p=0.0007; 0.09 [0.03-0.09] and 0.10 [0.10-0.10], respectively, p=0.0422; CD8: 0.06 [0.02-0.09] and 0.10 [0.04-0.10], respectively, p=0.0703; 0.06 [0.03-0.10] and 0.10 [0.05-0.11], respectively, p=0.0533) (**Figure 2D**).

Finally, at T0, pwMS showed higher plasma levels of APRIL, BAFF, and CD40L compared to HD (APRIL: 13296 [8890-18759] and 833 [220-3042] pg/ml, respectively, p<0.0001; BAFF: 6330 [2015-16971] and 429.3 [154-631] pg/ml, respectively, p<0.0001; CD40L:111275 [75329-132373] and 26664 [12457-55197] pg/ml, respectively, p<0.0001) (**Figure 2E**). Otherwise, at T1, only plasma levels of BAFF were still higher in pwMS compared to HD (9616 [1204-13922] and 594 [143-1097] pg/ml, respectively, p=0.0022) (**Figure 2E**). No significant differences in plasma levels of APRIL and CD40L were observed (**Figure 2E**).

### The longitudinal evaluation of humoral and specific T-cell response, and B-cell activating and survival factors

The two-point longitudinal evaluation of humoral and T-cell response, as well as B-cell activating and survival factors was performed in 16 pwMS (female/male: 11/5; 42 [34-49] years) and 9 HD (female/male: 7/2; 44 [33-53] years).

**Figure 3:**
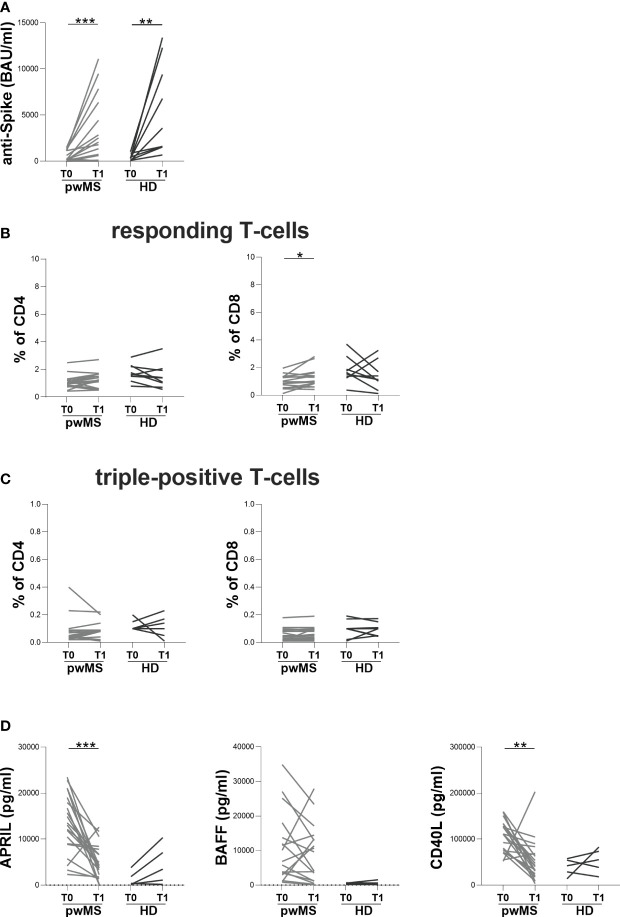
Longitudinal evaluation of humoral and specific T-cell response in pwMS and HD. **(A)** The longitudinal evaluation of anti-S antibody titers in pwMS and HD. **(B)** The evaluation of responding CD4+ and CD8+ T-cells in pwMS and HD. **(C)** The evaluation of triple-positive CD4+ and CD8+ T-cells in pwMS and HD. **(D)** The longitudinal evaluation of plasma levels of APRIL, BAFF and CD40L. APRIL, A-proliferation inducing ligand; BAFF, B-cell activating factor; CD40L, CD40 ligand; pwMS, people with multiple sclerosis; HD, healthy donors; T0, before third dose of vaccine; T1, after two months from third dose of vaccine. *p < 0.05; **p < 0.01; ***p < 0.001.

At T1, both pwMS and HD showed an increase in anti-S antibody titers compared to T0 (pwMS: 1930 [245-5895] and 198.5 [81-1140] BAU/ml, respectively, p=0.0006; HD: 3590 [1575-10850] and 320 [124-662] BAU/ml, respectively, p=0.0039) (**Figure 3A**). Concerning specific T-cell response, an increase in the percentage of responding CD8+ T-cells in pwMS was observed (1.17 [0.86-1.56] and 1.00 [0.60-1.33], respectively, p=0.0136) (**Figure 3B**). Conversely, no differences in the percentages of responding CD4+ T-cells neither in the percentages of triple-positive T-cells in both pwMS and HD were found (responding CD4+ T-cells: 1.23 [0.60-1.63] and 1.03 [0.80-1.28], respectively; triple-positive CD4+ T-cells: 0.09 [0.03-0.09] and 0.05 [0.03-0.09], respectively; triple-positive CD8+ T-cells: 0.06 [0.03-0.10] and 0.05 [0.02-0.09], respectively) (**Figure 3B, C**).

In pwMS, the evaluation of B-cell activating and survival factors showed a significantly reduction in plasma levels of APRIL and CD40L at T1 compared to T0 (APRIL: 4173 [1926-7510] and 13296 [8890-18759] pg/ml, respectively, p=0.0001; CD40L: 41546 [21284-68397] and 111275 [75329-132373] pg/ml, respectively, p=0.0012) (**Figure 3D**). Conversely, in pwMS no differences in plasma levels of BAFF were observed (**Figure 3D**) as well as in the longitudinal evaluation of APRIL, BAFF and CD40L plasma levels in HD (**Figure 3D**).

### Two-point cross-sectional evaluation of humoral and T-cell response, and B-cell activating and survival factors in pwMS stratified according to DMTs

**Figure 4:**
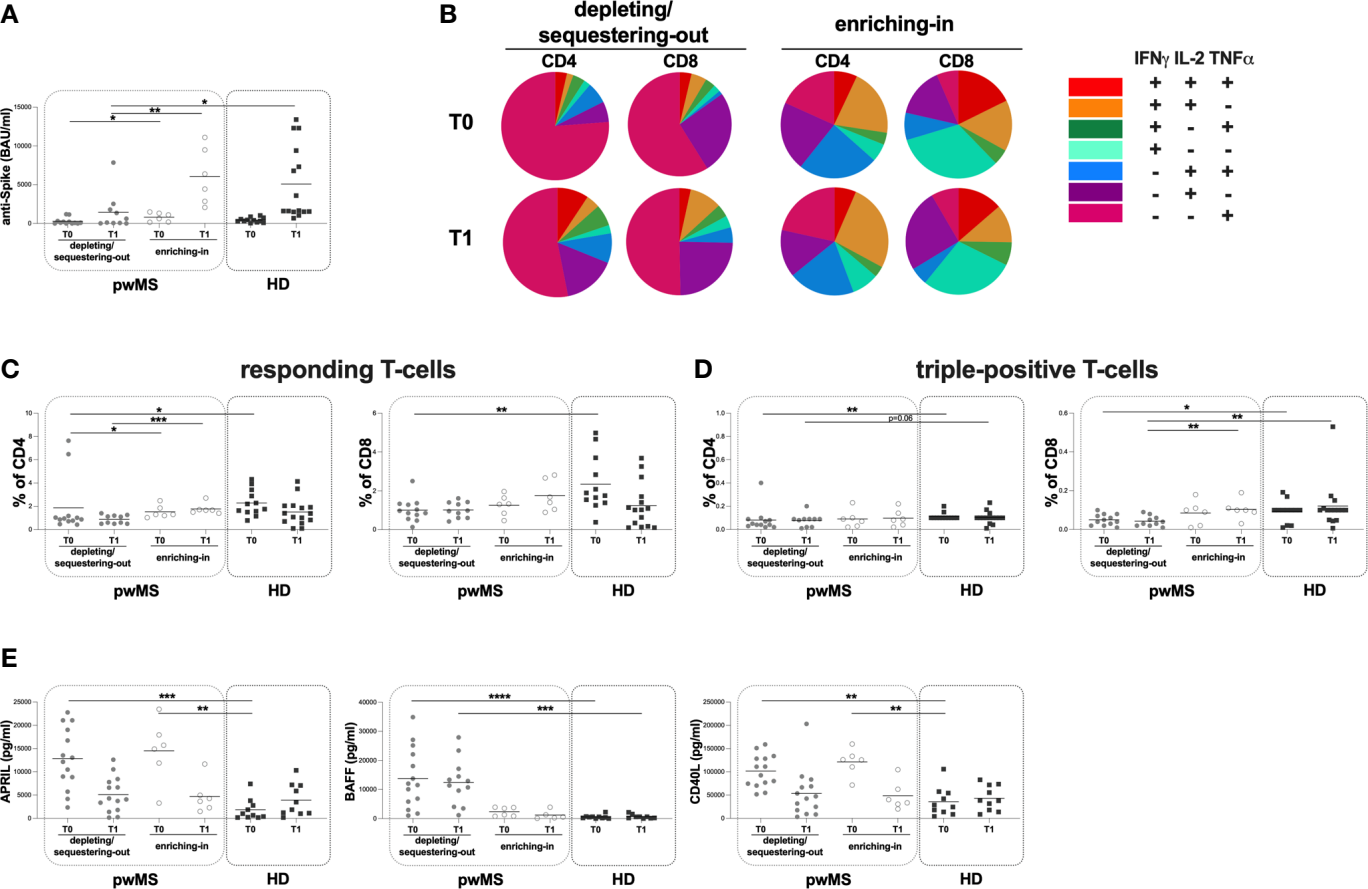
Cross-sectional evaluation of humoral and specific T-cell response is pwMS subgroups and HD, and overview of cytokine-producing T-cells. **(A)** The evaluation of anti-S antibody titers at two time-points: T0 and T1 in the depleting/sequestering-out and the enriching-in subgroups, and HD. **(B)** Overview of intracellular IFNγ, IL2 and TNFα production by CD4+ and CD8+ T-cells at T0 and at T1 in the depleting/sequestering-out and the enriching-in subgroups. **(C)** Evaluation of responding CD4+ and CD8+ T-cells in pwMS subgroups and HD. **(D)** Evaluation of triple-positive CD4+ and CD8+ T-cells in pwMS subgroups and HD. **(E)** Evaluation of plasma levels of APRIL, BAFF and CD40L in the depleting/sequestering-out and the enriching-in subgroups, and HD. APRIL, A-proliferation inducing ligand; BAFF, B-cell activating factor; CD40L, CD40 ligand; pwMS, people with multiple sclerosis; HD, healthy donors; T0, before third dose of vaccine; T1, after two months form third dose of vaccine. *p < 0.05; **p < 0.01; ***p < 0.001; ****p < 0.0001.

Stratifying pwMS according to DMTs, at both T0 and T1, a lower anti-S antibody titer in the depleting/sequestering-out compared to the enriching-in subgroup was found (T0: 100 [1-292] and 871 [175-1360] BAU/ml, respectively, p=0.0410; T1: 370 [50-1975] and 5410 [2655-9893] BAU/ml, respectively, p=0.0047) (**Figure 4A**). Moreover, only at T1, the depleting/sequestering-out subgroup showed a lower anti-S antibody titer compared to HD (370 [50-1975] and 1660 [1520-9400] BAU/ml, respectively, p=0.0244) (**Figure 4A**). No significantly differences in anti-S antibody titers between the enriching-in subgroup and HD ween seen (**Figure 4A**).

Interestingly, at both time-points, in the enriching-in subgroup, a heterogeneous cytokine production was observed (**Figure 4B**). Conversely, an unusual T-cell subset distribution in the depleting/sequestering-out subgroup at both time-points was found (**Figure 4B**). Specifically, the depleting/sequestering-out subgroup showed a higher percentage of IFNγ-IL2-TNFα+ CD4+ T-cells compared to the enriching-in one at both time point, although at T1 the differences were not statistically significant (T0: 1.31 [0.38-3.76] and 0.20 [0.08-0.32], respectively, p=0.0187; T1: 0.51 [0.23-2.40] and 0.27 [0.16-0.41], respectively). Likely, a higher percentage of IFNγ-IL2-TNFα+ CD8+ T-cells in the depleting/sequestering-out subgroup compared to the enriching-in one at both time-points was observed (T0: 0.72 [0.45-0.82] and 0.04 [0.02-0.06], respectively, p=0.0007; T1: 0.52 [0.33-0.74] and 0.06 [0.01-0.55], respectively, p=0.0077).

At both T0 and T1, a lower percentage of responding CD4+ T-cells in the depleting/sequestering-out compared to the enriching-in subgroup was seen (0.92 [0.73-1.15] and 1.30 [1.16-2.01], respectively, p=0.0394; 0.85 [0.50-1.22] and 1.68 [1.48-1.96], respectively, p=0.0004) (**Figure 4C**). No differences in the responding CD8+ T-cell percentages between the depleting/sequestering-out and the enriching-in subgroups were observed (T0: 0.91 [0.60-1.32] and 1.31 [0.75-1.72], respectively; T1: 0.97 [0.60-1.41] and 1.54 [1.00-2.70], respectively) (**Figure 4C**). Conversely, at T1, a lower percentage in triple-positive CD8+ T-cells in the depleting/sequestering-out compared to the enriching-in subgroup was observed (0.04 [0.02-0.07] and 0.10 [0.08-0.13], respectively, p=0.0082) (**Figure 4D**).

Finally, at T0, a lower percentage of responding T-cells in the depleting/sequestering-out subgroup compared to HD was found (CD4: 0.92 [0.73-1.15] and 1.98 [1.52-3.29], respectively, p=0.0116; CD8: 0.91 [0.60-1.32] and 1.82 [1.42-3.48], respectively, p=0.0049) (**Figure 4C**). A both T0 and T1, a lower percentage in triple-positive CD4+ T-cells in the depleting/sequestering-out subgroup compared to HD was seen, although not statistically significant at T1 (T0: 0.05 [0.03-0.09] and 0.10 [0.10-0.10], respectively, p=0.0024; T1: 0.09 [0.02-0.09] and 0.10 [0.10-0.10], respectively, p=0.0645) (**Figure 4D**).

Moreover, at both T0 and T1, lower percentages of triple-positive CD8+ T-cells in the depleting/sequestering-out subgroup compared to HD was found, although not statistically significant at T0 (T0: 0.05 [0.02-0.08] and 0.10 [0.04-0.10], respectively, p=0.0588; T1: 0.04 [0.02-0.07] and 0.10 [0.05-0.11], respectively, p=0.0048) (**Figure 4D**).

At both time-points, no differences in the plasma levels of APRIL and CD40L between the depleting/sequestering-out and the enriching-in subgroups were observed (APRIL T0: 12603 [8077-19540] and 15312 [9740-11670] pg/ml, respectively; T1: 4173 [1706-7957] and 3965 [2082-6646] pg/ml, respectively; CD40L T0: 101855 [73681-129802] and 125967 [101016-140260] pg/ml, respectively; T1: 45043 [15469-72595] and 36953 [28036-73177] pg/ml, respectively) (**Figure 4E**). Otherwise, at T0 and T1, a higher plasma level of BAFF in the depleting/sequestering-out compared to the enriching-in subgroup was seen (T0: 11768 [5094-228865] and 2412 [836.30-3807] pg/ml, respectively, p=0.0064; T1: 12146 [5409-164509] and 504.90 [163.30-2578]pg/ml, respectively, p=0.0023) (**Figure 4E**).

At T0, a higher plasma level of APRIL in both the depleting/sequestering-out and the enriching-in subgroups compared to HD was observed (the depleting/sequestering-out: 12603 [8077-19540] and 832.70 [220.10-3042] pg/ml, respectively, p=0.0005; the enriching-in: 15312 [9740-11670] and 832.70 [220.10-3042] pg/ml, respectively, p=0.0017) (**Figure 4E**). At T1, no differences were observed (**Figure 4E**). Otherwise, at T0 and T1, a higher plasma level of BAFF in the depleting/sequestering-out subgroup compared to HD was observed (T0: 11768 [5094-228865] and 429.30 [154.20-630.80] pg/ml, respectively, p <0.0001; T1: 12146 [5409-164509] and 594.10 [142.50-1097] pg/ml, respectively, p=0.0004) (**Figure 4E**). No differences in the plasma level of BAFF between the enriching-in subgroup and HD were found (**Figure 4E**). Finally, at T0 a higher plasma level of CD40L in both the depleting/sequestering-out and the enriching-in subgroups compared to HD was observed (the depleting/sequestering-out: 101855 [73681-129802] and 26664 [12457-55197] pg/ml, respectively, p=0.0013; the enriching-in: 125967 [101016-140260] and 26664 [12457-55197] pg/ml, respectively, p=0.0012) (**Figure 4E**). No differences in plasma level of CD40L between pwMS subgroups were seen (**Figure 4E**).

### Two-point longitudinal evaluation of humoral and T-cell response, and B-cell activating and survival factors in pwMS stratified according to DMTs

**Figure 5:**
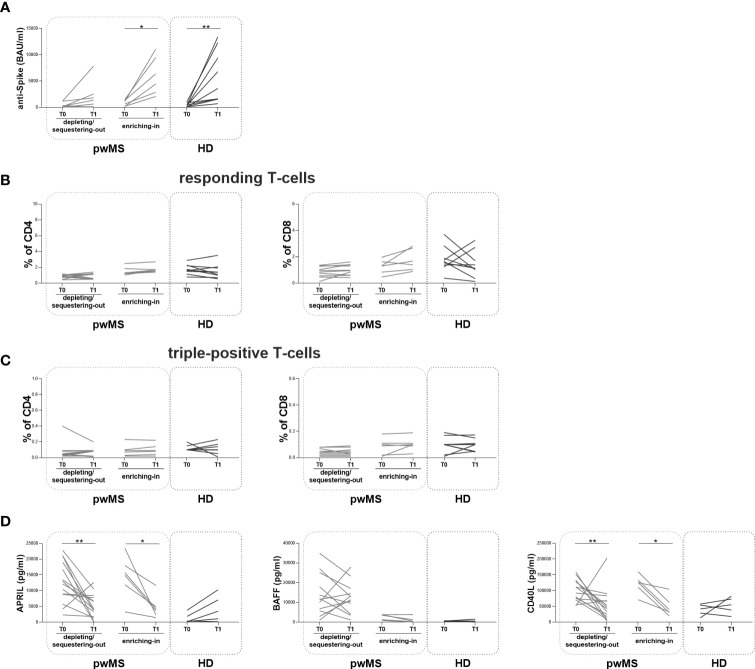
Longitudinal evaluation of humoral and specific T-cell response pwMS subgroups and HD. **(A)** The longitudinal evaluation of anti-S antibody titers between T0 and T1 in the depleting/sequestering-out and in the enriching-in subgroups compared to HD. **(B)** The evaluation of responding CD4+ and CD8+ T-cells in pwMS subgroups. **(C)** The evaluation of triple-positive CD4+ and CD8+ T-cells in the depleting/sequestering-out and in the enriching-in subgroups. **(D)** The longitudinal evaluation of plasma levels APRIL, BAFF and CD40L. APRIL, A-proliferation inducing ligand; BAFF, B-cell activating factor; CD40L, CD40 ligand; pwMS, people with multiple sclerosis; HD, healthy donors; T0, before third dose of vaccine; T1, after two months from third dose of vaccine. *p < 0.05; **p < 0.01.

At T1, the longitudinal evaluation of anti-S antibody titer showed a significant increase in the enriching-in subgroup compared to T0 (5410 [2655-9893] and 871 [175.30-1360] BAU/ml, respectively, p=0.0313) (**Figure 5A**).

In both pwMS subgroups, no differences in the percentages of responding and triple-positive T-cells were observed (**Figure 5B, C**). Finally, at T1, a significant reduction in plasma levels of APRIL and CD40L in both pwMS subgroups compared to T0 was observed (the depleting/sequestering-out subgroup: APRIL: 4173 [1706-7957] and 12603 [8077-19540] pg/ml, respectively, p=0.0031; CD40L: 45043 [15469-72595] and 101855 [73681-129802] pg/ml, respectively, p=0.0245; the enriching-in subgroup: APRIL: 3965 [2082-6646] and 15312 [9740-19322] pg/ml, respectively, p=0.0313; CD40L: 36953 [28036-73177] and 125967 [101016-140260] pg/ml, respectively, p=0.0313) (**Figure 5D**).

## Discussion

In this observational, monocentric, and prospective study, we investigated the immunogenicity before and after the third dose of BNT162b2 mRNA vaccine in pwMS under different DMTs, evaluating both humoral and specific T-cell response as well as B-cell activating and survival factors and comparing the obtaining findings with a control group.

In line with other studies involving different pwMS (8, 32–34), the first main result of our study was that pwMS treated with DMTs develop a positive humoral immune response to the mRNA vaccine, which does not differ significantly from that observed in HD. Moreover, an increase in humoral response in pwMS following the third dose of BNT162b2 mRNA vaccine was seen. However, as reported by Sabatino et al. (32), humoral response to SARS-CoV-2 vaccine appears to be influenced by different DMTs mechanism of action. Indeed, in our study, pwMS belonging to the depleting/sequestering-out subgroup (including alemtuzumab-, cladribine-, fingolimod- and ocrelizumab-treated) showed a significantly lower humoral response to vaccination when compared to HD and to the enriching-in subgroup (natalizumab-treated). This is in agreement with several published studies in which a pattern of low humoral response to SARS-CoV-2 vaccination, with respect to healthy subjects, has been previously reported, mainly for patients receiving B-cell depleting drugs (35–37) and fingolimod (32, 38). Even though the depleting/sequestering-out subgroup displayed lower anti-Spike antibody titers, most patients had near-normal total anti-Spike IgG levels, while only few did not seroconvert. This particular phenomenon could be due, as already proposed by Hausler et al., to an incomplete depletion of B-cells by anti-CD20 treatment, that mainly act on circulating B-cells, leaving a smaller number of these cells that may persist in secondary lymphoid tissues (32, 39).

Although the first line of protection against SARS-CoV-2 includes pre-existing antibodies, induced by vaccination or infection, great safeguard can also be attributed to the T-cell response (40, 41). Indeed, as shown by Agrati et al. (42), in immunocompetent subjects the BNT162b2 mRNA vaccine is able to elicit a coordinated spike-specific T-cell response characterized by a production of all Th1 cytokines, with IFNγ correlating with both TNFα and IL2. Given that, we performed in pwMS a broad characterization of the functional profiles of specific T-cells, comparing the obtaining findings with HD. One strength of our study was the evaluation of all possible combination of intracellular expression of IFNγ, IL2 and TNFα by T-cells. T-cells that produce more than one of the three cytokine of interest have been considered as to be important in response to viral infections, including influenza (43, 44). Moreover, in convalescent COVID-19 patients this polyfunctional cytokine profile has been observed suggesting a possible rapid recall response (45–47).

In our study, pwMS showed lower percentages in responding and triple-positive T-cells compared to HD. Interestingly, when stratifying pwMS according to DMTs, lower percentages in responding and triple-positive T-cells were seen mainly in the depleting/sequestering-out subgroup. Different results have been reported in pwMS, with an extensive T-cell response in natalizumab-treated patients, an adequate T-cell response in ocrelizumab-treated and an impaired one in fingolimod-treated ones (2, 21, 32, 48, 49). The lower T-cell mediated response to vaccination that we observed in the depleting/sequestering-out subgroup is in accordance with published studies in which a reduction or even absence of adaptive cellular response has been reported in patients treated with fingolimod (50, 51). An explanation to this phenomenon could be the mode of action of fingolimod itself, that may result in trapping relevant T-cells in secondary lymphoid tissues blocking *in vitro* responses (51).

Moreover, in our study, pwMS included into the depleting/sequestering-out subgroup showed a higher percentage of IFNγ-IL2-TNFα+ T-cells at both time-points, compared to the enriching-in subgroup and HD in which a more heterogeneous cytokine profile was observed. These data suggest an inferior quality of response in pwMS included into the depleting/sequestering-out subgroup. This is in line with results from Picchianti-Diamanti et al. (52), showing a production of only one cytokine by T-cells in fragile patients and suggesting a potential dysfunction in T-cell response in frail subjects.

Lastly, due to B-cell involvement in vaccination immune response and in mounting an immunological memory (28), we evaluated plasma concentration of B-cells activating and survival factors, BAFF, APRIL and CD40L (25, 53, 54). Higher plasma levels of BAFF, APRIL and CD40L were seen at baseline in pwMS when compared to HD, difference that lasted in the depleting/sequestering-out and in the enriching-in subgroups. This is in accordance with some studies in which higher plasma levels of the three cytokines are reported in pwMS when compared to HD, due to their involvement in worsening of MS pathogenesis and in its regulation (55–58). A reduction over-time in APRIL and CD40L plasma concentration was seen in pwMS and the two subgroups, supporting their involvement in immune response to vaccination (59–61). However, no differences in plasma levels of BAFF over-time were observed. Being involved in B-cell survival and promotion, BAFF receptor expression is critical in enhancing an immune response and antiviral immunity (62). These results suggest that, event tough it is lower than healthy subjects’, a humoral response is still elicited in pwMS.

Our study has some limitations such as the small sample size and the extremely heterogeneous pwMS DMTs included. On the other hand, an alemtuzumab-treated patients was included into the study, a treatment difficult to include due to the reduced use of this drug.

## Conclusion

In summary, our data underline that the third dose of BNT162b2 mRNA vaccine provides additional benefit to pwMS. However, according to DMT mechanism of action, pwMS should be addressed toward the use of pre-exposure monoclonal antibodies, that have been proved to be effective in mounting an adequate humoral response (63), and to other therapeutic strategies to prevent SARS-CoV-2 infection, when necessary. T-cell and antibody titer testing of patients under certain DMTs may allow a more individualized counselling of their infection risk. Finally, an understanding of T-cell quality dynamic is needed to determine the best vaccination strategy and in general the capability of immune response in pwMS under different DMT.

## Data availability statement

The original contributions presented in the study are included in the article/supplementary material. Further inquiries can be directed to the corresponding author.

## Ethics statement

The study was approved by Ethics Committee of Policlinico Umberto I, Sapienza University of Rome (protocol numbers 0062/2022). The patients/participants provided their written informed consent to participate in this study.

## Author contributions

MZ, MT, MC, AC: Designed the study. MZ, FD, MG, ET, AN, VP: Performed laboratory testing, analyzed data, performed statistical analysis, and wrote the manuscript. MC, AC, CM, ML: Assisted in designing the study. MZ, AC, MC, ML: Discussed results and critically revised the manuscript. MT, LM, PP, FC, VB, AG: Provided clinical samples and clinical data. MZ, MC, AC, ML: Discussed results, read, and revised the manuscript. All authors contributed to the article and approved the submitted version.

## Funding

This study was supported by Sapienza University of Rome, PhD in "ADVANCES IN INFECTIOUS DISEASES, MICROBIOLOGY, LEGAL MEDICINE AND PUBLIC HEALTH SCIENCES".

## Acknowledgments

The authors would like to thank all participants for their participation in this study.

## Conflict of interest

The authors declare that the research was conducted in the absence of any commercial or financial relationships that could be construed as a potential conflict of interest.

## Publisher’s note

All claims expressed in this article are solely those of the authors and do not necessarily represent those of their affiliated organizations, or those of the publisher, the editors and the reviewers. Any product that may be evaluated in this article, or claim that may be made by its manufacturer, is not guaranteed or endorsed by the publisher.
